# UWB Monitoring System for AAL Applications

**DOI:** 10.3390/s17092092

**Published:** 2017-09-12

**Authors:** Jerzy Kolakowski, Vitomir Djaja-Josko, Marcin Kolakowski

**Affiliations:** Institute of Radioelectronics and Multimedia Technology, Warsaw University of Technology, Nowowiejska 15/19, 00-665 Warsaw, Poland; v.djaja-josko@ire.pw.edu.pl (V.D.-J.); m.kolakowski@ire.pw.edu.pl (M.K.)

**Keywords:** UWB, localization systems, AAL, behaviour monitoring, MEMS

## Abstract

Independent living of elderly persons in their homes requires support that can be provided with modern assistive technologies. Monitoring of elderly persons behaviour delivers valuable information that can be used for diagnosis and detection of health problems as well as triggering alerts in emergency situations. The paper includes a description of the ultra wideband system developed within Networked InfrasTructure for Innovative home Care Solutions (NITICS) Active and Assisted Living (AAL) project. The system can be used as a component of AAL platforms. It delivers data on users localization and has a fall detector functionality. The system also provides access to raw measurement results from Microelectromechanical Systems (MEMS) sensors embedded in the device worn by the monitored person. These data can be used in solutions intended for elderly person’s behaviour investigation. The system was investigated under laboratory conditions as well as in home environment. The detailed system description and results of performed tests are included in the article.

## 1. Introduction

World population is getting older. In Europe, according to EC report on aging [1], the number of people aged 65 and over is expected to grow from 18% of the population (in 2013) to 28% in 2060. Maintaining quality of life for all these people becomes a demanding task. One of the main points of Active and Assisted Living (AAL) programme strategy [2], is the promotion of independent living of elderly persons in their homes for as long as possible.

Elderly person’s independent living is almost impossible without help of others. The majority of effort connected with the age-related care in Europe is made by family, friends and healthcare workers (80% according to [2]). Therefore, the solutions supporting caretakers as well as caregivers are highly demanded. The caretakers’ burdens can be lessened with Assistive Technologies (AT) defined as “any devices or systems that allow an individual to perform a task they would otherwise be unable to do or increase the ease and safety with which the task can be performed” [3].

According to [4,5], the main objectives of AT are:

- to help with everyday activities (e.g., moving between places, rehabilitation, help in intellectual activities);
- to gather and process health related data (e.g., temperature, blood pressure, sugar level);
- to monitor elderly persons (e.g., detection of health endangering events, behaviour analysis); and
- to support socialization and leisure activities (e.g., helping to make contacts with other people, playing games).

Recently, the great progress made in Information and Communication Technologies (ICT) encourages many investigators to search for AAL solutions in this field. The UWB (Ultra Wideband) monitoring system presented in the article also exploits ICT. It is a part of the platform developed within NITICS AAL project [6]. Although it is mainly intended for monitoring tasks, some of its functions can also be used for support elderly persons in everyday activities. Moreover, it can be used as a source of data in other AAL solutions.

**Figure 1:**
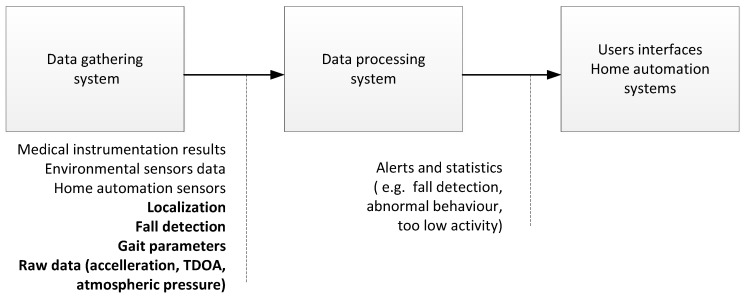
Data processing in Assistive Technologies (AT) platform.

Majority of AT platforms are built in accordance with the general architecture presented in Figure 1. Data gathering system contains devices delivering data to be processed. Environmental sensors (e.g., ambient temperature and humidity sensors), medical instruments (e.g., blood pressure meters, pulse oxymeters, and blood glucose meters), user interfaces (e.g., touch screens and microphones) and UWB monitoring system belong to this group. The UWB monitoring system provides information on person’s localization, fall detection alerting and gait related parameters as well as raw measurement results.

Data processing system uses gathered data to evaluate monitored person’s behaviour (e.g., physical activity, detection of deviations from routine activities, and detection of dementia patient wandering) and to control home automatics (e.g., lighting, door locks, and air condition system). At the last stage, data processing results are exploited. Information is sent to caretakers over user interfaces or is consumed by devices included in home automation system.

Localization and tracking is a key functionality of the presented monitoring system. Positioning accuracy of the system should be high enough to retrieve the route passed by the monitored person. It is essential in certain AAL applications, e.g., in detection of wandering occurring in case of elderly dementia patients. In addition to an information on visited room, the system should detect a situation when a person is approaching certain objects e.g., kitchen stove. It might be helpful in assessing monitored patient’s behaviour. For such purposes sub-meter positioning accuracy is usually sufficient.

Many technologies can be used to determine person localization. Laser scanners, ultrasound transceivers, radio based systems and infrared or visible light systems are only a few examples of solutions that can be used for this purpose. The up-to-date survey of positioning technologies can be found in [7].

From above technologies radio solutions seem to have most advantages. Due to through the wall propagation the quantity of nodes comprising positioning system infrastructure can be significantly reduced. There are many radio technologies used for localization. Unfortunately, the most popular solutions based on Bluetooth Low Energy (BLE) and WiFi devices do not provide sufficient localization accuracy in indoor environment. The UWB technology was chosen as it allows for positioning with sub-meter accuracy. UWB positioning systems are more accurate and precise than other commonly used narrowband radio technologies. Wide signal bandwidths, ability to resolve signal components in time domain and time of arrival measuring techniques are the reasons behind their better performance.

The developed system integrates UWB with Microelectromechanical Systems (MEMS) technology to improve localization accuracy and reliability. Embedding MEMS sensors in the localization system tag also allows gathering information on person’s body movements. Fall detection and gait characterization can be made with collected data.

The article describes an ultrawideband monitoring system for AAL applications. Section 2 contains a description of current achievements in the field and comparison of proposed solution to other developments. Section 3 includes the system description, and presents implemented functions, system architecture and particular devices. In Section 4, algorithms used to process raw measurement results into demanded data are presented. Section 5 is dedicated to system tests.

## 2. Related Works

The number of publications dealing with use of different assistive technologies is enormous. They range from simple devices intended for performing one function, e.g., fall detection to complex systems of much broader functionalities. Therefore, related works analysed in this section deal mainly with systems utilizing wearable sensors and providing similar functionalities: localization, fall detection and extraction of gait related parameters. Most of these solutions use wireless transfer of results from sensors.

### 2.1. Persons Tracking

Localization of elderly persons in AAL applications is a subject of many papers. In majority of solutions, Wireless Local Area Network (WLAN) and Wireless Personal Area Network (WPAN) networks are used. However, there are also works concerning technologies such as UWB, ultrasonic and Bluetooth Low Energy (BLE). Use of inertial sensors or some combination of mentioned technologies is also described by some authors.

Xie et al. [8] presented a work related to WiFi based indoor localization using RSSI measurements performed by a smartphone. Three different data classification methods were presented; however, localization accuracy was limited to detecting a room in which the smartphone was located. Xu et al. [9] have also proposed WiFi and Received Signal Strength (RSS) measurement based localization. They introduced new method, supporting selection of access points providing noisy or redundant information and therefore improving accuracy.

In [10], a BLE based system is described. The results allow assigning the person to the particular room. Monitoring of the activities of daily living of the elderly with BLE beacons is presented in [11,12]. Recorded signal levels were fused with results from smartphone’s inertial sensors. Presented approach involves usage of the iPhone 5’s accelerometer, compass and its ability to measure level of signals coming from BLE tags and integrates them using Kalman Filter. Presented results, with the average localization estimation error below 1 m, are quite promising. However, tests were performed in situations where the phone was carried in a hand in front of the tester, which explicitly oriented it and allowed for easier step and orientation estimation.

Similar system based on iBeacon technology is presented in [13]. iBeacon devices are deployed in the tracking area. RSS measurements are performed by the device worn by the person. However, position calculation is performed server-side as the gathered are immediately sent to the central unit and is not stored locally.

There are also several works involving RFID-based indoor localization. Huang et al. [14] presented an approach that combines wearable MEMS sensors with active RFID tags serving as beacons deployed in the vital positions of the area covered by the localization system. MEMS sensors are attached to the person at their lower limbs (two per leg) and waist (one sensor), which allows for estimation of the posture (which can be distinguished as sitting, standing, squatting, supine and prone) and gait-based position calculation. Active RFID tags are used for the position correction when person would get in their read-range. Achieved accuracies are in range of 1–2 m. Another approach was presented by Wang et al. [15]. The proposed system consists of active RFID readers distributed in the tracking area. Monitored elderly person wears the RFID tag and the smartphone and is localized when coming in the range of the readers.

There are also reports of localization using ultrasonic signals. Charlon et al. [16] have presented a telemetry system combining two wireless technologies—ultrasonic and 802.15.4. Both signals are used to calculate ToF (Time of Flight) between the worn tag and the beacons mounted in the area covered by the system. Gathered data are further used to estimate position of the person and movement data, such as gait speed or trajectories.

Accuracies achieved in localization systems based on narrowband radio interfaces are usually low, as can be seen in cited works. Reconstruction of movement trajectory necessary for detection of dementia patient wandering or determination of time spent in particular places at home may be difficult. UWB localization systems seem to be a more suiting solution to these problems. Comparison of UWB and RFID based localization systems can be found in [17]. Conclusions in that paper confirm much better positioning accuracy of UWB system.

For several years, UWB positioning systems have been successfully implemented in many areas in industry and commerce. Unfortunately, AAL applications of such systems are relatively rare. Some of the UWB indoor localization research is focused on the UWB radar technology as in [18]. Experiments consisting in analysis of movements of six dementia patients with the use of commercial positioning system [19] are described in [20]. Gathered location traces allowed for evaluation of patient state, detection of relations between patients and provided insight into their habits. The drawback of the solution is a very high cost of the system. Zhao et al. [21] presented comparison between the Ubisense UWB system and Cricket ultrasonic system.

There are a few articles describing positioning systems built from Decawave chips used also in the monitoring system presented in the article. Contigiani et al. [22] describe tests of two UWB IEEE802.15.4a based commercial localization systems in retail environment. In both solutions Decawave chips were used. Although the supermarket environment is different from home environment, obtained results proved that such systems can be used for efficient trajectory recording.

### 2.2. Fall Detection

Solutions found in the literature usually present one of two different approaches to elderly person fall detection. The first one consists in embedding sensors and devices into environment where the person lives, while the second one is based on wearable sensors.

Sensors located in elderly person environment are mainly intended for investigation of elderly person behaviour, activity recognition and fall detection. Radar sensors or cameras are typically used for this purpose. Some of the proposed systems use typical video cameras, which is perceived controversial due to possible privacy violations. Therefore, depth cameras [23], which allow preserving privacy while maintaining high system efficiency, gain more recognition. Another solution detects a fall by analysis of its effects: sounds and vibrations caused by a person’s body hitting the ground. An example of the system fusing measurements of both types of signals can be found in [24]. The common drawback of ambient sensors is their number required to cover all rooms in an elderly person’s home.

The wearable sensors are an interesting alternative allowing for system infrastructure reduction but they have to be worn by the person, which is less convenient for the monitored person. MEMS sensors, which recently have strongly developed, are becoming cheaper and more accurate, making them the most popular choice for wearable fall detection systems. Especially tri-axial accelerometers with possible support of compass and gyroscope are getting a lot of attention in recent works. Bagala et al. [25] have presented thorough investigation of the accelerometer-based fall detection with an emphasis on the real-life falls. Dias et al. [26] have also described fall detection monitoring using wearable sensors. Furthermore, they have proposed a ZigBee based communication system for wireless transmission of the results.

Wang et al. [27] have presented a fall-detection system based on the wearable MEMS sensors integrated with the active RFID technology used for easy localization of the patient in the case of falling down. Huang et al. have proposed similar solution in [14].

However, MEMS sensors are not the only solution for devices capable of detecting falls or even detecting patients posture. Wang and Gu [28] propose a wearable RFID system that combines wearable sensors and RFID tags for real time activity recognition. Passive RFID tags, which are light, maintenance free and can be sewn into clothes are used. RFID reader is attached to the person’s body and it senses the RFID tags. As BAN (Body Area Network) propagation model is quite sophisticated and strongly dependent on the body position, Wang and Gu propose a method utilizing analysis of multiple radio patterns coming from multiple RFID tags attached to different body parts. That way, specific radio pattern combinations may be related to specific body positions and activities.

### 2.3. Conclusions

The article authors do not have solutions integrating UWB positioning and movement analysis functionalities, in which accelerometers are used not only for movement evaluation (e.g., fall detection) but also for improving positioning accuracy. UWB solutions are mainly dedicated to position determination but not fall or activity detection. They are far less spread than BLE or WiFi based systems, however their key features such as devices’ small sizes and high positioning accuracy might allow them to gain ground among the indoor localization systems. Moreover, most commercially available systems as well as most experimental systems still utilize wired synchronization between the devices [19,29,30], which makes them hard to install in most home environments.

None of presented systems provides complex solution, integrating precise and accurate ultrawideband localization with the wearable MEMS sensors allowing for fall detection, gait analysis and ability to provide data that can be further analysed in terms of elderly monitoring and assisting. In comparison to presented state of the art solutions, novelty of the developed solution consists in:

- integration of UWB positioning and movement analysis functionalities;
- a positioning algorithm fusing step parameters and positioning results;
- using UWB radio packets not only for localization but also for data transfer from tags;
- wireless synchronization of the positioning system infrastructure nodes that provides easier system installation (reduction of cabling); and
- reduction of the number of sensors in the tag resulting in better tag’s energy efficiency.

## 3. UWB Monitoring System

### 3.1. System Requirements

During system design phase, a set of requirements concerning functionality and design was formulated. The system should be able to reliably track an elderly person in his/her home, provide data for person’s gait characterization and include fall detector functionality. Tracking should be performed with sub-meter accuracy so that a retrieval of a passing route would be possible. Due to relatively small movement velocity, positioning rate of a few times per second should be sufficient. The elderly person will be equipped with a small wearable device attached to a belt, a lanyard or attached to the wrist with Velcro strap. The system infrastructure should be unobtrusive, and cables between infrastructure devices should be eliminated.

The above requirements had an impact on the system design. The accuracy requirement forced the use of UWB signals for positioning. The need for unobtrusivity resulted in wireless system infrastructure. Synchronization signals and measurement results are sent over UWB or WiFi links. The number of sensors in the wearable device was limited to accelerometer and barometer because of their low power consumption. The gyroscope due to high consumed current was not included in the design.

Finally, the changes and the need for reliability forced to develop a new positioning algorithm fusing measurement results performed with UWB receivers and results obtained from wearable device sensors.

### 3.2. System Architecture

**Figure 2:**
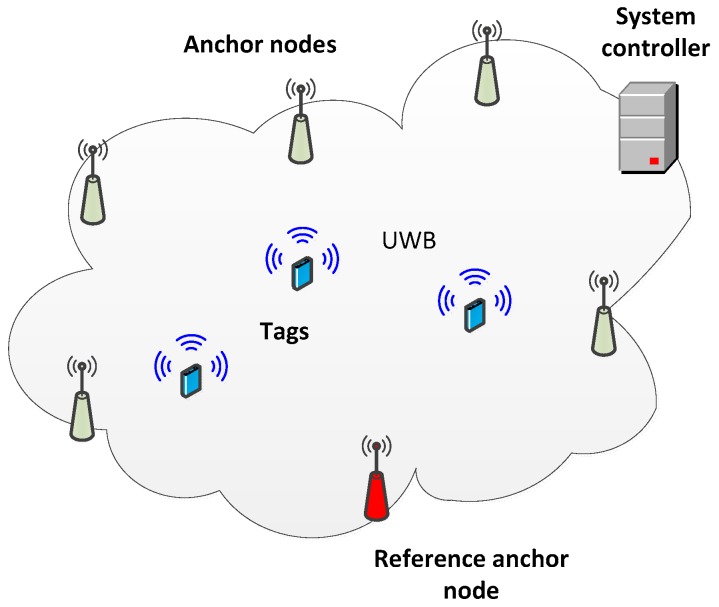
Monitoring system architecture.

The functional architecture of UWB monitoring system is presented in Figure 2. The system is composed of anchor nodes installed in the building and tags worn by the monitored persons. The tags are equipped with sensors for acceleration and atmospheric pressure measurements and UWB transmitters. The UWB radio link conforms to the IEEE 802.15.4a standard [31]. Transmitted packets contain results of measurements carried out in the tag.

Two types of anchor nodes are used in the system:

- standard nodes responsible for reception of packets from tags and reference nodes and for packets time of arrival measurement; and
- reference anchor nodes equipped with a precise temperature compensated oscillators, which are responsible for reference signals broadcasting. Reference signals enable correction of measurement errors resulting from the anchors oscillators frequencies difference. The correction process is described in Section 4.1.

Anchor nodes send results of performed measurements and data retrieved from tags’ packets to the system controller over WiFi links. There tracked persons’ locations are calculated.

### 3.3. Tag

**Figure 3:**
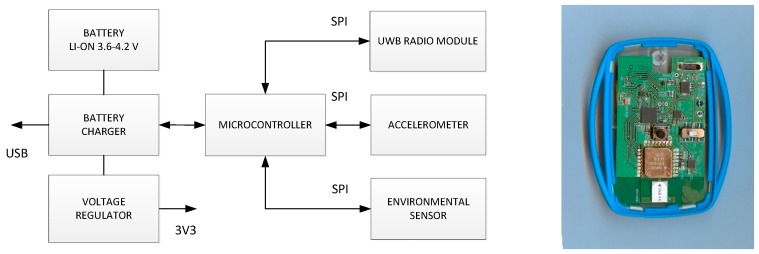
Tag architecture and assembled tag.

The system tag is a wearable device, thus should be small, light and energy efficient. Therefore, only two MEMS sensors, accelerometer and atmospheric pressure meter, were embedded. The tag architecture is shown in Figure 3. The tag is controlled by MSP430FR5739 (Texas Instrument) microcontroller, providing low current operation modes. The use of FRAM memory allowed significantly reducing current consumption. Communication with sensors and the radio module is conducted over chip’s SPI interfaces.

The tag is equipped with two MEMS sensors, accelerometer (BMA280) and environmental sensor (BME280)—both from Bosch Sensortec. The BMA280 is a tri-axial device able to measure acceleration in two ranges, ±2 g to ±16 g with 14 bit resolution. The second MEMS sensor (BME280) is able to measure temperature, humidity and atmospheric pressure.

DWM1000 module (from Decawave) comprising DW1000 chip [32] and UWB ceramic antenna was used as a UWB transmitter. The signals were transmitted in 5th channel (6.5 GHz), and signal bandwidth was close to 500 MHz. To increase transmission range, a preamble of 2048 symbols was used. Data rate was equal to 110 kbit/s. After each transmission, the module is moved to sleep mode.

The tag is supplied from one 3.7 V LiIon 400 mAh battery. All components are supplied via BQ24071 battery charger chip used for battery current control during discharge and charge processes. The micro USB connector was used for external power supply connection. Current consumptions of particular tag components are included in Table 1.

**Figure 4:**
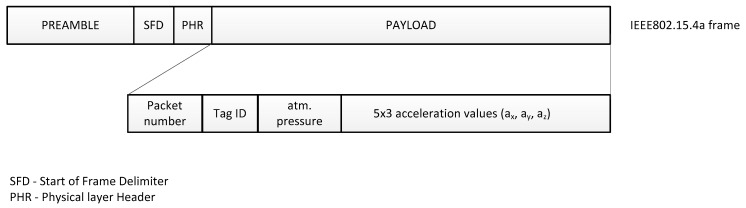
Tag’s frame payload.

The sampling rate of the employed sensors is different. Accelerometer is sampled every 32 ms, barometer every 160 ms. Measurement results are placed in the IEEE801.15.4a packet payload and transmitted with 160 ms period. The transmitted frame content is shown in Figure 4. Total frame length equals to 35 bytes.

### 3.4. Anchor Nodes

**Figure 5:**
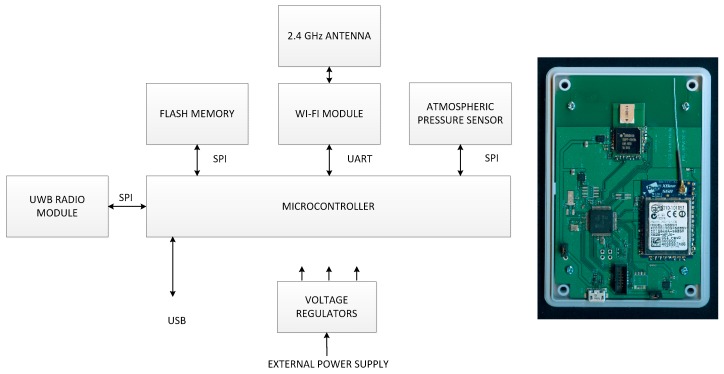
Anchor node/reference node architecture and assembled anchor node board.

Designed anchor nodes and reference node share the same hardware design presented in Figure 5. The only difference is the use of custom built UWB radio module including DW1000 chip, TCXO oscillator and UWB antenna in the reference anchor node. All anchor nodes are equipped with the TIVA series TM4C123GH6PM, ARM Cortex-M4F microcontroller. It is the main processing unit, and controls other peripheral devices. For communication with the system controller, a XB2B XBee WiFi module connected to the 2.4 GHz antenna is used. UWB radio module is used for reception of packet from the tag and for communication with other anchors. Devices are also equipped with flash memory and a BMP183 atmospheric pressure sensors that provides reference pressure measurements. Anchors are powered from the external power supply.

The principal task of the anchor node is a measurement of UWB packets time of arrival. The internal functions of DW1000 transceiver are used for this purpose. The chip measures time of arrival with 15.65 ps resolution. Moreover, it is able to initiate packet transmission at precisely predefined moments. Both functionalities are crucial in implementation of transmission schedule presented in Section 4.1.

**Figure 6:**
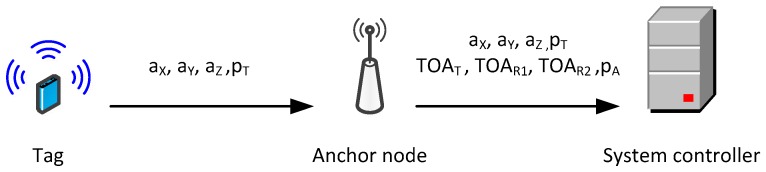
Measurement results transmitted in the system.

WiFi interface is used to transmit measurement results to the system controller (Figure 6). Anchor node retransmits results obtained from the tag, acceleration components (a_X_, a_Y_, a_Z_) and atmospheric pressure p_T_. It also transmits atmospheric pressure measured by the anchor (p_A_) and time of arrival (TOA) values measured for packets sent by the tag (TOA_T_) and the reference anchor node (TOA_R1_ and TOA_R2_).

The anchor node configuration data (e.g., network addresses, identifiers, and radio link parameters) are stored in the node’s flash memory. Modification of flash content is possible over WiFi or USB links.

### 3.5. System Controller

**Figure 7:**
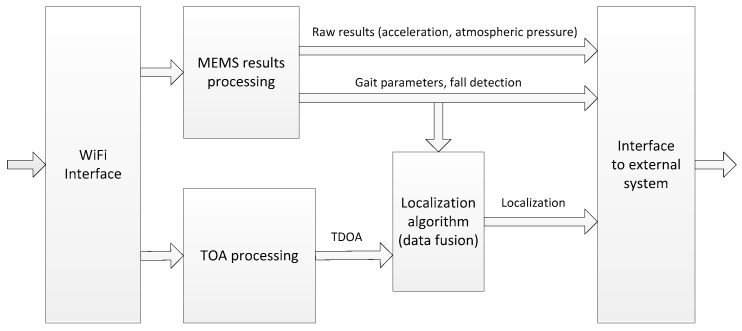
Data processing in the system controller.

The system controller is a computer that performs a central role in the monitoring system. The data flow and data processing in the controller are illustrated in Figure 7. Data from packets received over WiFi link are passed to two processing blocks. In the first one, MEMS results (acceleration and pressure) are used for gait parameters evaluation and fall detection. Results of analysis and raw measurement data are available to external systems via interface. Access to raw data allow for implementation of other gait recognition or fall detection algorithms.

Received time of arrival values are processed in the TOA processing function (Section 4.1). Resulting TDOA values along with extracted gait parameters are the input data to the positioning algorithm described in Section 4.3. Coordinates of monitored person are available to external systems over interface block. USB or Ethernet interfaces can be used for that purpose.

## 4. Algorithms

### 4.1. TDOA Determination and Correction Method

**Figure 8:**
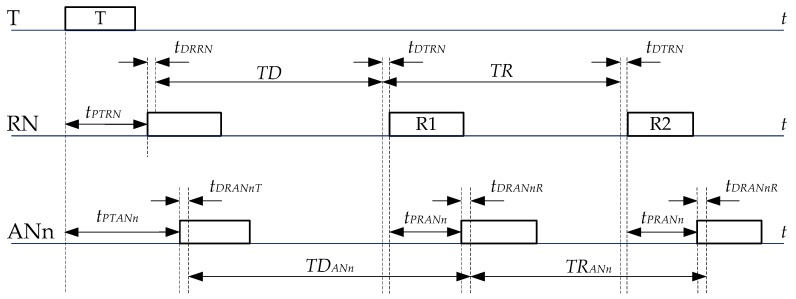
Exemplary transmission scheme.

Proposed system architecture implies a specific transmission scheme of packets between the devices. An exemplary transmission scenario involving the tag (T), the anchor node (ANn) and the reference anchor node (RN) is presented in Figure 8.

Transmission is initialized by the tag, which sends first packet (T), and received by anchor nodes, which record the time of reception of the packet. The reference anchor, after waiting for a fixed period (*TD*), transmits its own packet (R1). It is received by anchor node ANn. Moreover, the reference anchor sends another packet (R2) after some predefined time *TR*, which is received by ANn as well.

Times *t_PTRN_* and *t_PTANn_* are unknown propagation times between the tag and the reference node and anchor node, respectively. Time *t_PRANn_* is the propagation time between the reference node and the anchor node, which is known since anchors are stationary at fixed positions. *TD* is the period of time between the reception of the tag’s packet in the reference node and transmission of its own packet; *TR* is reference period; and *TD_ANn_* and *TR_ANn_* are real time intervals between the received packets in the anchor node ANn.

In Figure 8, propagation time between the tag and the anchor node ANn is as follows:

(1) $$t_{PTANn}=TD-TD_{ANn}+t_{PTRN}+t_{PRANn}+t_{DRRN}+t_{DTRN}+t_{DRANnR}-t_{DRANnT}$$

where *t_DRRN_* and *t_DTRN_* are, respectively, unknown internal reception and transmission delays introduced in the reference node (they are strictly related to the DW1000 chip); and *t_DRANnT_* and *t_DRANnR_* are internal reception delays introduced in the anchor node ANn, when measuring time of arrival of the packets coming from the tag and the reference node, respectively.

Anchor node measures *TD_ANn_* period. Result of measurement (*TDM_ANn_*) depends on the internal clock oscillator frequency offset.

(2) $$TDM_{ANn}=TD_{ANn}\left(1+\epsilon_{ANn}\right)$$

where $\epsilon_{ANn}$ is relative clock frequency error in anchor node ANn.

To reduce oscillator impact on measurement results, the reference period measurement is performed in the anchor. The measured reference period equals to:

(3) $$TRM_{ANn}=TR_{ANn}\left(1+\epsilon_{ANn}\right)=TR\left(1+\epsilon_{ANn}\right)$$

Therefore,

(4) $$TD_{ANn}=\frac{TR}{TRM_{ANn}}TDM_{ANn}$$

None of the internal reference node delays have impact on the reference period measurements because packets related to the reference period are sent from the same node.

Clock frequency errors affect also generation of the reference period in the reference anchor:

(5) $$TR=TR_{p}\left(1+\epsilon_{R}\right)$$

where *TR_p_* is predefined reference period value, set in the reference anchor node; and $\epsilon_{R}$ is relative clock frequency error in the reference anchor node.

Reference period measurement performed in the anchor node is used.

Finally, time interval between packets, with respect to measured values, is as follows:

(6) $$TD_{ANn}=\frac{TR_{p}\left(1+\epsilon_{R}\right)}{TRM_{ANn}}TDM_{ANn}$$

Positioning algorithm implemented in the system utilizes TDOA values. TDOA value for the anchors ANn and ANm is equal to:

(7) $$TDOA_{mn}=t_{PTANm}-t_{PTANn}$$

From Equation (1), the *TDOA_mn_* equation can be written as:

(8) $$TDOA_{mn}=TD_{ANm}-\;TD_{ANn}\;+t_{Pmn}+t_{DELmn}$$

where

(9) $$t_{Pmn}=t_{PRANn}-t_{PRANm}$$

(10) $$t_{DELmn}=t_{DRANnR}-t_{DRANnT}-\;t_{DRANmR}+t_{DRANmT}$$

Component *t_Pmn_* corresponds to propagation time difference between reference anchor node and ANn and ANm anchors. Delays related component *t_DELmn_* groups internal anchors delays. Its value is close to zero if the delays are similar. Finally, the measured TDOA is equal to:

(11) $${\mathrm{TDOA}}_{\mathrm{mn}}=\frac{TR_{p}\left(1+\epsilon_{R}\right)}{TRM_{ANm}}TDM_{ANm}-\;\frac{TR_{p}\left(1+\epsilon_{R}\right)}{TRM_{ANn}}TDM_{ANn}\;+t_{\mathrm{Pmn}}+t_{\mathrm{DELmn}}$$

Accuracy of TDOA determination mainly depends on accuracy of propagation times between the reference anchor and other anchor nodes determination and on internal anchors’ delays. Impact of the relative reference clock frequency error is small because difference between *TM_ANm_* and *TM_ANn_* periods is usually in the order of tens of nanoseconds.

### 4.2. Gait Parameters Determination

**Figure 9:**
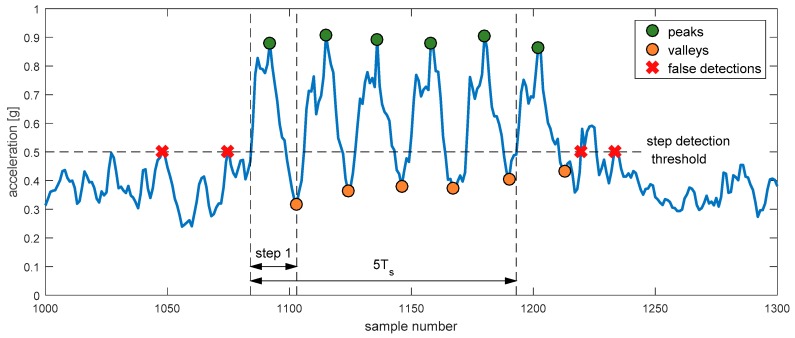
Acceleration measured for a sequence of six steps. Detected peaks and valleys are marked with green and red dots respectively. False step detections, which would occur for a simple threshold algorithm, are marked with red crosses.

The system allows determining basic gait parameters of the localized person, which are step period, step length and heading. These parameters are computed based on acceleration measurements and previously calculated localizations. The acceleration is measured at a rate higher than rate of position determination which depends on TDOA availability. Each packet sent by the tag carries a few acceleration results. It allows determining step parameters with greater accuracy. The accelerometer used in the tag device measures acceleration in three perpendicular axes. The tag does not contain any additional sensors such as gyroscopes and magnetometers so it is not possible to accurately translate measured accelerations to system’s coordinates. Therefore, gait parameters are determined based on acceleration magnitude. Gait parameters evaluation algorithm is illustrated in Figure 9.

Gait parameters evaluation is conducted with a few samples delay. It allows using more advanced step detection algorithm than a simple threshold detection. The proposed step detection procedure begins with locating peaks and valleys in the registered acceleration signal. To lower the probability of false step detection, only the peaks above certain level are considered. The algorithm analyses samples preceding localized peak. A sample for which the fixed step detection threshold was exceeded is treated as the moment of step’s start. The first located valley following the analysed peak is treated as step’s end. Such analysis allows determining step’s start more precisely and minimize the number of false step detection, which would occur for basic threshold detection algorithms (marked with red crosses).

The times of step’s start and end are recorded and used to determine gait parameters. The step period is calculated every time the step is detected and is averaged with the previous value using moving average. Step length and direction of movement are calculated based on the number of steps taken and the covered distance estimated from the computed person’s localizations.

**Figure 10:**
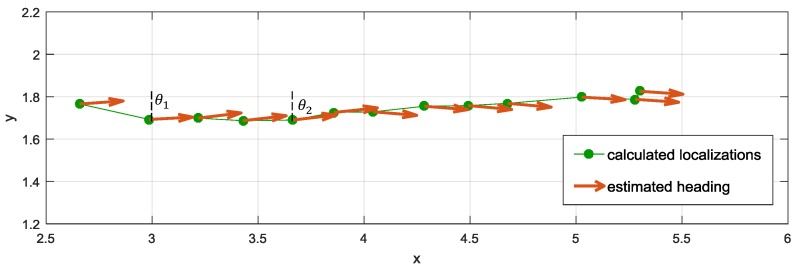
The illustration of heading estimation.

The idea of heading estimation is presented in Figure 10. Heading is estimated based on a few previously computed localizations using linear regression. The proposed approach assumes that the trajectory of persons movement for the analysed sets of points is close to the straight line. Given that the system’s update rate is relatively high, and elderly persons do not tend to rapidly change movement direction, such approximation seems to be justified. Linear regression method allows calculating the parameters of a line, which represents movement trajectory. In the algorithm, instead of those parameters, heading direction is defined with *θ*, which is an angle between the obtained line and *Y*-axis.

### 4.3. Positioning Algorithm

**Figure 11:**
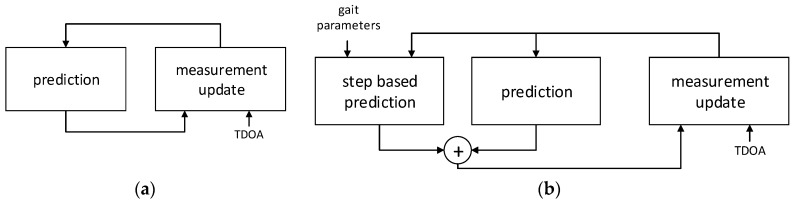
Localization algorithms workflow scheme: (**a**) basic Extended Kalman Filter utilizing TDOA measurement results; and (**b**) localization algorithm used in the system.

The positioning algorithm used in the system combines TDOA values obtained with the system infrastructure with the results of acceleration measurements conducted by the tag. The workflow of the algorithm is presented in Figure 11.

In the basic EKF algorithm [33], a localized person is modelled as a system, which state is described with his coordinates and velocity.

(12) $$x_{k\;}=\;{\left[x\;y\;v_{x}v_{y}\right]}^{T}$$

The single iteration of EKF (Figure 11a) consists of two phases: prediction, where a current state of a system is predicted based on the value obtained in the previous iteration and persons movement model typically comprising basic equations of motion and measurement update; and correction, where predicted value is corrected based on TDOA results obtained from the system.

In the prediction phase of the EKF algorithm, person movement is modelled as a uniform linear motion with constant velocity between the analysed points. The first prediction phase is described by the following equations:

(13) $${\hat{x}}_{k\left(-\right)}^{\left(1\right)}=F{\hat{x}}_{k-1\left(+\right)}$$

(14) $$P_{k\left(-\right)}^{\left(1\right)}{\;=\;\mathrm{FP}}_{k-1\left(+\right)}F^{T}{+Q}_{k}^{\left(1\right)}$$

where ${\hat{x}}_{k\left(-\right)}^{\left(1\right)}$, $P_{k\left(-\right)}^{\left(1\right)}$ and ${\hat{x}}_{k-1\left(+\right)},\;P_{k-1\left(+\right)}$ are predicted state vector value and state vector from previous filter iteration with corresponding covariance matrices; F is state transition matrix containing equations of motion; and $Q_{k}^{\left(1\right)}$ is process noise covariance matrix, the value of which is chosen in accordance with Discrete White Noise Acceleration Model (DWNA) [34].

The algorithm used in the system (Figure 11b) is a modification of EKF. The major novelty with respect to basic EKF is the introduction of another, parallel prediction phase. In the additional step based prediction block, localized person’s movement is modelled as a sequence of steps, which is described by step period *Ts*, step length *Ls* and heading direction *θ*. Gait parameters determination is described in Section 4.2. Equations comprising step based prediction phase are as follows:

(15) $${\hat{x}}_{k\left(-\right)}^{\left(2\right)}={\hat{x}}_{k-1(+)}+{\left[sin\theta \;cos\theta \;0\;0\right]}^{T}L_{s}\Delta T/T_{s}$$

(16) $$P_{k\left(-\right)}^{\left(2\right)}{\;=\;P}_{k-1\left(+\right)}{+Q}_{k}^{\left(2\right)}$$

where ${\hat{x}}_{k\left(-\right)}^{\left(2\right)}$ is the state vector predicted based on gait parameters analysis and $P_{k\left(-\right)}^{\left(2\right)}$ is a corresponding covariance matrix, ΔT is the position refresh period equal to 160 ms. Localized person’s predicted position is obtained by adding to the previously computed position a translation resulting from the step taken by the user. If during gait parameter analysis no step was detected, it is assumed that the person is still and no translation is added.

State vector values obtained from both prediction phases are combined in the way similar to that in Kalman Filter. The covariances of predicted vectors are analysed and the most probable state vector value is estimated [35]. The obtained predicted state vector is then corrected with TDOA values at measurement update phase.

(17) $$h_{k}\left(x_{k}\right)=\left[\begin{array}{ccc} {\mathrm{TDOA}}_{1}\left(x_{k}\right) & \cdots & {\mathrm{TDOA}}_{n}\left(x_{k}\right) \end{array}\right]$$

(18) $$K_{k}{=P}_{k\left(-\right)}H_{k}^{T}{\left(H_{k}P_{k\left(-\right)}H_{k}^{T}{+R}_{k}\right)}^{-1}$$

(19) $${\hat{x}}_{k\left(+\right)}={\hat{x}}_{k\left(-\right)}+K_{k}(z_{k}-h_{k}({\hat{x}}_{k\left(-\right)}))$$

(20) $$P_{k\left(+\right)}=\left({I-K}_{k}H_{k}^{T}\right)P_{k\left(-\right)}$$

The corrected state vector is a linear combination of the predicted value and a measurement innovation, which is a difference between TDOA measurement results $z_{k}$ and corresponding TDOA values calculated for the predicted tag location $h_{k}({\hat{x}}_{k(-)})$. The weight, with which measurement results are taken into account, is specified by Kalman gain $K_{k}$, which is calculated based on measurement matrix $H_{k}$, which is a linearization of $h_{k}\left(x_{k}\right)$ and measurement covariance matrix $R_{k}.$ The number of used TDOA results varies and depends on their availability. The corrected state vector value is a final result of the algorithm and is used as input data in next iterations.

### 4.4. Fall Detection Algorithm

**Figure 12:**
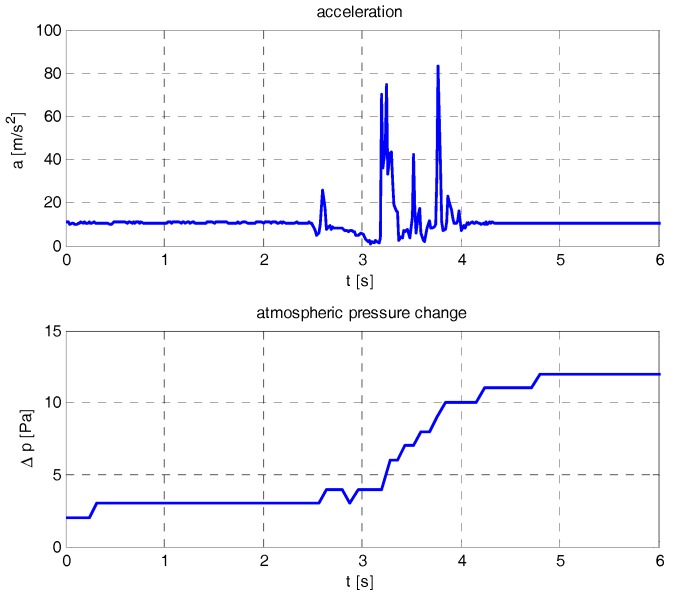
Acceleration and atmospheric pressure variations during the fall (experimental data).

Fall detection algorithm utilizes information on changes of acceleration and atmospheric pressure recorded by sensors embedded in the tag. Algorithm development was preceded by the preliminary experiments performed using the test setup described in Section 5.2. A typical acceleration magnitude and atmospheric pressure changes are shown in Figure 12. Similar signals were also presented by other researchers (e.g., [25,36]).

Before the fall, the acceleration magnitude is close to 10 m/s^2^. The first peak (at 2.5 s) is caused by the test manikin release mechanism. Next, a free-fall starts. During this phase, acceleration magnitude decreases. Hitting the ground results in significant acceleration peak, followed by a few secondary peaks. Starting free fall phase is also seen in the atmospheric pressure graph. After the fall, when manikin lies down, pressure stabilizes at higher value, acceleration returns to the value before the fall.

**Figure 13:**
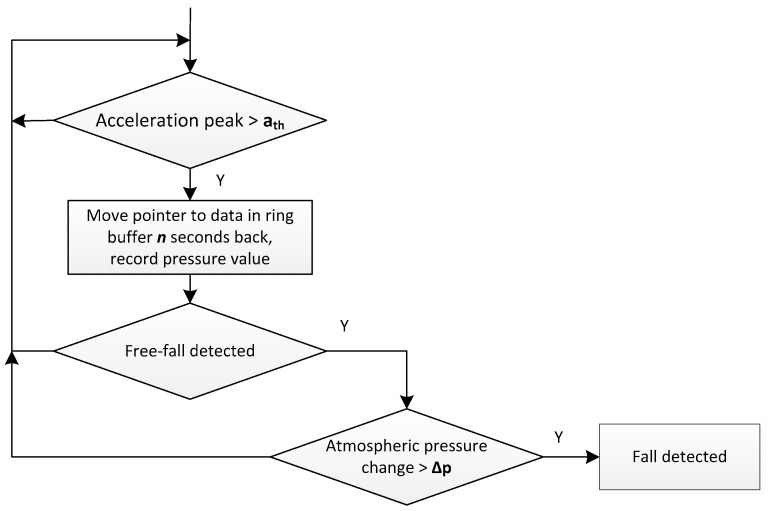
Fall detection algorithm.

Fall detection algorithm is presented in Figure 13. It continuously operates on acceleration and pressure data stored in ring buffers. Free fall detection is achieved by the cross correlation of acceleration and a function, the shape of which corresponds to acceleration changes during free fall.

The following algorithm parameters were used during algorithm tests:

- acceleration magnitude threshold a_th_ was set to 30 m/s^2^;
- ring buffer shift (*n*) equal to 1.5 s; and
- pressure change Δp equal to 10 Pa.

## 5. System Tests

### 5.1. Positioning System Tests

#### 5.1.1. Test Site

Test campaign was carried out in the Institute of Radioelectronics and Multimedia Technology laboratory rooms and two fully furnished flats. The results collected in one of the flats, as more representative for considered system application, are presented in this section. The flat is composed of small rooms, and is located in panel building constructed of pre-fabricated, pre-stressed concrete. The test system consisted of seven anchor nodes and one reference anchor. The aim of the test was to determine positioning and tracking accuracy. Two kinds of tests were carried out: tests in static positions and tracking of a moving person.

#### 5.1.2. Static Tests

The test scenario consisted in placing the tag on the tripod at different test points and recording a few hundred calculated positions at each location. Positioning error (bias) is defined as the distance between test point and the point corresponding to median *x* and *y* coordinate values. Circular Error Probability (CEP), referring to the radius of a circle in which assumed percentage of results is located, was chosen as a precision measure. The 68th and 95th percentiles were used for CEP calculation.

**Figure 14:**
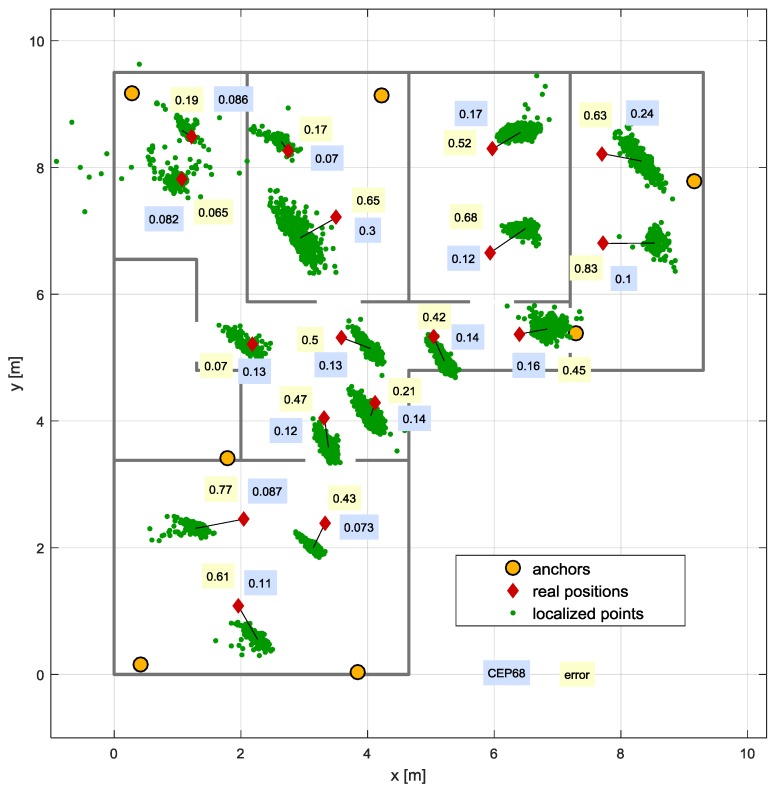
Positioning errors and Circular Error Probabilities (CEP) at particular test points.

Results of positioning are presented on the flat layout (Figure 14). Calculated locations are marked with small green dots. Red diamonds representing the test points are linked with points corresponding to calculated mean positions (coordinates are equal to mean *x* and *y* values). Bias expressed in meters is shown on the yellow background. CEP values for 68th percentile (also in meters) are presented on blue background.

**Figure 15:**
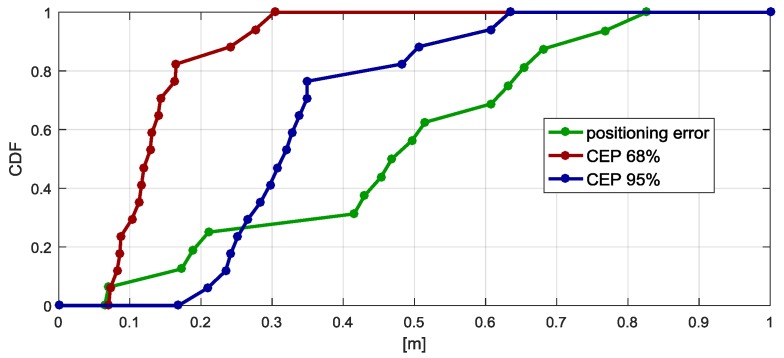
Empirical Cumulative Distribution Functions (CDF) of positioning errors and CEP coefficients.

The empirical Cumulative Distribution Functions (CDF) corresponding to positioning errors and CEP coefficients are shown in Figure 15. Positioning bias is lower than 0.65 m in 80% of test points. The precision for 80% of the analysed points is high: CEP does not exceed 0.16 m.

The obtained results demonstrate that quality of positioning results depends not only on the availability of the signals, but also on their quality. The use of radio signals allows placing anchor nodes in different rooms. Therefore, in most tests, the system worked in Non Line Of Sight (NLOS) conditions. In such case, the signal travelled along different paths, penetrated through walls, and underwent multiple reflections, refractions, and diffractions. All these phenomena greatly affected the received signal as well as the positioning accuracy.

In the considered flat, the signal had to pass a number of walls made of reinforced concrete. Big signal attenuation resulted in precision deterioration. Significant shifts in obtained positions were observed in cases where direct path components of transmitted signals were blocked, e.g., by mirrors (two wardrobes with doors fitted with large mirrors were installed in the flat).

Another factor having an impact on system performance is location of anchor nodes, which was a compromise between requirements resulting from localization algorithm needs and flat interior design.

#### 5.1.3. Person Tracking

**Figure 16:**
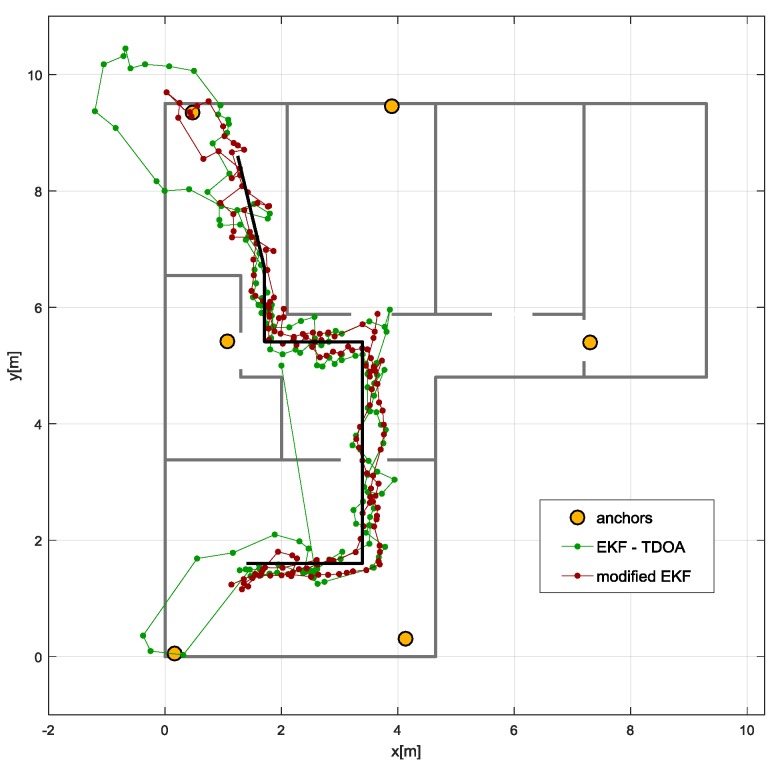
Tracking results obtained with Extended Kalman Filter (EKF) using Time Difference of Arrival (TDOA) values and proposed algorithm exploiting fusion of TDOA and gait parameters (modified EKF).

The tag was attached to the belt worn by the person. The route was marked on the floor, and the person walked along the predefined path. Tracking error is characterized by the distance from obtained point to the closest point of the path. Tracking errors are presented as the empirical Cumulative Distribution Function (CDF). Results of tracking the person moving from one of rooms to the kitchen and back are shown in Figure 16.

**Figure 17:**
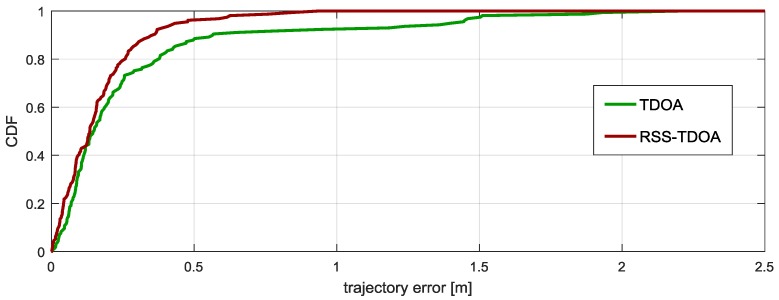
Empirical CDF of tracking error for all tested algorithms.

To check proposed algorithm (modified EKF) efficiency, obtained results were compared with results from algorithm utilizing only TDOA values (EKF-TDOA). The proposed solution resulted in much better path reconstruction. It can especially be seen at the end of the paths, where the localized person was turning around. The use of the novel algorithm allowed suppressing negative effects caused by rapid changes of anchor visibility during person’s turnaround. Observations are confirmed by Empirical CDF graphs shown in Figure 17. More than 95% of obtained locations show deviation from the line smaller than half a meter.

### 5.2. Fall Detection Tests

Fall detection test consisted of the use of a rescue manikin [37] as a substitute to falling person. The manikin’s 66 kg weight is distributed according to human weight distribution chart. Due to articulated joints, its behaviour during the fall is similar to a human’s. Manikins have been used by other researchers for fall detection testing [24,38]. The recorded acceleration signals are similar to real life signals (e.g., [36,39]). Use of the manikin for fall investigation is an interesting alternative to tests performed by (usually young) volunteers. It does not require additional safety measures like mattresses or head and limbs shields, which have an impact on obtained results.

**Figure 18:**
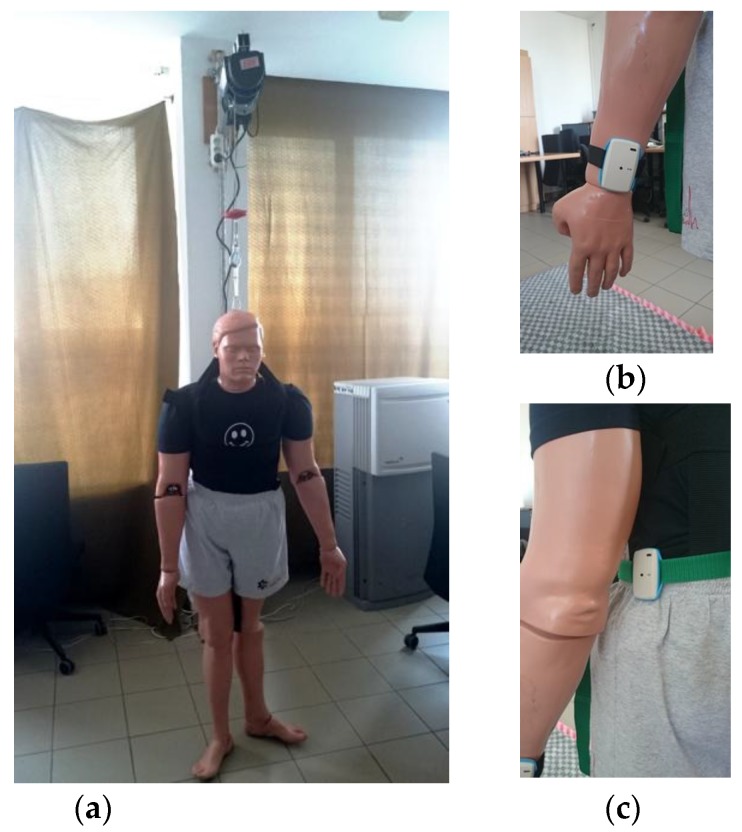
Manikin on the test site (**a**); and tags attached to the: wrist (**b**); and waist (**c**).

During tests, the system tags were attached to different parts of manikin’s body. The manikin was lifted using electric winch and then released by burning out polyamide cord. The photograph of the test site and exemplary attachment of system tags are shown in Figure 18.

**Figure 19:**
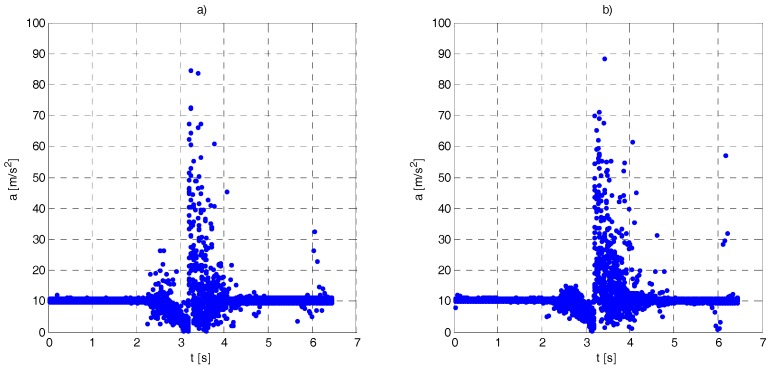
Acceleration magnitude versus time during 20 falls: (**a**) tag attached to the manikin waist; and (**b**) tag attached to the wrist.

Each fall resulted in change of acceleration magnitude and increase in atmospheric pressure by several Pa. Exemplary changes of acceleration magnitude during the fall recorded for several falls are shown in Figure 19.

During the test campaign, the falls from standing and kneeing positions were investigated. In both cases, the acceleration and atmospheric pressure graphs were similar (the pressure change was lower). Performed test scenarios correspond to cases where the person is falling limply. Similar acceleration and pressure changes were observed during real falls reported in [23,34,36].

**Figure 20:**
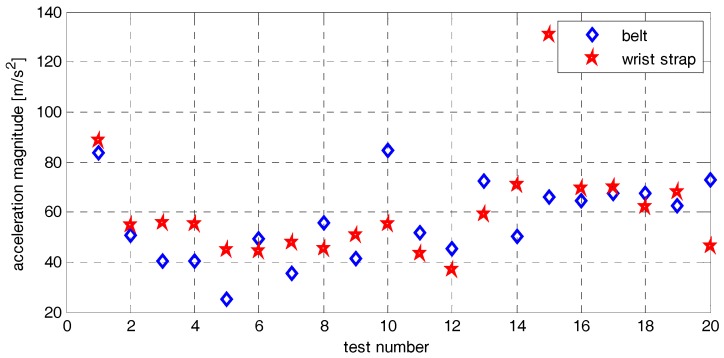
Acceleration magnitude maxima achieved during tests.

The system operated correctly during the test, all sensors results were recorded. The fall detection algorithm was tested using 40 data records. Only in one case, it did not detect the fall. Analysis or recorded samples shown that in one case the acceleration did not reach the required 30 m/s^2^ threshold. Acceleration magnitude maxima are shown in Figure 20.

## 6. Conclusions

The article contains a description of the UWB system intended for elderly persons monitoring. The system delivers information on person’s localization, parameters of his gait and information on detected falls. These data can be used in algorithms intended for evaluation of elderly person behaviour or detection of illness symptoms, e.g., wandering of dementia patients. The system also provides access to raw data that can be useful for development of customized algorithm.

There are a few novel solutions implemented in the system. Wireless communication between anchor nodes and wireless anchors synchronization simplify system installation and exploitation. The system tag energy consumption is reduced due to selection of energy efficient MEMS sensors. A new positioning algorithm fusing acceleration and TDOA was proposed.

The system was built and tested. Localization functionalities were tested in static and dynamic conditions tests. Results obtained in a typical flat confirmed sub-meter accuracy and precision in both kinds of tests. Fall detection algorithm was tested at a specially designed site with the use of rescue manikin. It proved that the system allows detecting fall events with high certainty.

Results of system investigation proved that developed UWB monitoring system can be successfully used in AAL applications.

## Figures and Tables

**Table 1:** Tag’s components current consumption.

Chip	Mode	Current Consumption
MSP430FR5739	Active	177 μA
	Sleep (Low-Power Mode 3)	6.3 μA
BME280	Measurement	12.8 μA
	Sleep (Sleep mode)	0.1 μA
BMA280	Measurement	130 μA
	Sleep (Suspend mode)	2.1 μA
DWM1000	Transmission	70 mA
	Sleep	100 nA
